# Organizational and psychosocial environmental work factors associated with self-rated exhaustion disorder among municipal employees in rural northern Sweden

**DOI:** 10.3233/WOR-220225

**Published:** 2023-08-11

**Authors:** Sofia Asplund, Britt-Marie Lindgren, Sture Åström, Mattias Hedlund, Johan Åhlin

**Affiliations:** a Department of Nursing, Umeå University, Umeå, Sweden; b Department of Community Medicine and Rehabilitation, Umeå University, Umeå, Sweden

**Keywords:** Occupational health, occupational stress, public sector, social support, workload

## Abstract

**BACKGROUND:**

Research indicates that good organizational and psychosocial environments are vital to well-functioning workplaces and employee health. Working in the municipal sector and in the rural context may contribute to more health problems, poorer organizational and psychosocial work environments, and higher sick-leave rates.

**OBJECTIVE:**

The aim of this study was to explore organizational and psychosocial environmental work factors among municipal employees with or without self-rated exhaustion disorder (s-ED) in rural northern Sweden.

**METHODS:**

The Modern Work Life Questionnaire and the Self-Rated Exhaustion Disorder Scale were used among 1093 municipal employees.

**RESULTS:**

The results showed that there were significant differences between the s-ED and the non–s-ED group in all but one of the organizational and psychosocial environmental work factors. Various demands, i.e. quantitative, emotional, intellectual, and IT demands were some factors associated with the s-ED group. Social support, resources, and time for work and reflection were some factors associated with the non–s-ED group. Both the s-ED and the non–s-ED groups assessed significantly higher emotional demands and less resources compared to national reference values.

**CONCLUSION:**

Findings from this study are relevant to a better understanding what organizational and psychosocial work environmental work factor the employer need to pay extra attention to. Addressing risk and protective factors in the work environment could tribute to promote occupational well-being, preventing exhaustion disorder and long-term sick leave among municipal employees in rural northern Sweden.

## 1 Introduction

Organizational and psychosocial environmental risk factors in the workplace can exhaust employees’ mental and physical resources and are associated with ill health [1], and long-term sick leave [2]. Extensive work for a longer period of time can affect the work-life balance [3], and cause stress-related health problems [4]. According to one of the most commonly used occupational stress models, the job demands-resources (JD-R) model [5], employees’ health and well-being depend on a balance of positive and negative organizational and psychosocial factors (resources vs. demands) in the work environment. High demands can exhaust employee’s mental and physical resources, and therefore lead to health problems, and considered to be the main causes of burnout. In contrast, sufficient resources foster employee engagement and may buffer the impact of demands on stress. The JD-R model can be used to understand, explain, and make predictions about employee burnout, work engagement, and outcomes, and is considered a useful framework for monitoring the workplace [6–8].

Burnout can be defined as a ‘syndrome conceptualized as resulting from chronic workplace stress that has not been successfully managed’, included in the International Classification of Diseases 11th edition (ICD). Three symptoms are included; feelings of energy depletion or exhaustion, increased mental distance from one’s job or feelings of negativism or cynicism related to one’s job, and reduced professional efficacy. Burnout is classified as an occupational phenomenon and not a medical condition, bound to the occupational context, and not applied to describe experiences in other areas of life [9]. One of the most used definition of burnout was presented by Maslach and co-workers [10], who defined it as a syndrome of emotional exhaustion, depersonalization and reduced professional accomplishment, mainly afflicting employees in helping professions. Burnout is an unspecific term, with various definitions and of psychological origin, unlike exhaustion disorder (ED), which is a more specific term and a clearly defined clinical diagnosis. The Swedish Board of Health and Welfare introduced the medical diagnosis of (ED) to facilitate the classification of patients seeking health care due to exhaustion caused by prolonged stress. The stressors can be caused by work, private life, and often a combination of both. Exhaustion disorder was accepted as a formal diagnosis in the Swedish version of the 10th revision of the ICD (F43.8A). The diagnostic criteria involve exhaustion symptoms, which have developed in response to stressors, and existed for at least six months. Lack of mental energy, reduced initiative, reduced endurance, or prolonged recovery time after mental strain are also important elements. Other symptoms such as concentration difficulties or memory problems, reduced ability to manage demands, sleep disturbances, gastrointestinal symptoms are also present. The symptoms cause clinically significant suffering or reduced ability to function at work, socially, or in other important situations [11, 12]. Exhaustion disorder has been proposed as the most valid clinical equivalent of burnout [13]. Research has shown that the majority (93%) of patients who fulfilled the criteria for ED also scored clinical burnout [14]. Research has shown that patients with exhaustion disorder appear to consult their general practitioner numerous times with stress-related complaints in the years preceding their diagnosis [15].

In Sweden, a large proportion of sick leave can be linked to occupational stress, and its association with the organizational and psychosocial work environment has increased over time. Exhaustion disorder (ED) is a common cause of sick leave, which for this diagnosis often exceeds 6 months [16]. Job insecurity, low influence of work-related decisions, high effort for low reward, and lack of support are important organizational and psychosocial factors related to ED [17]. People with ED have reported private relationship conflicts to be almost as important as work demands and usually attribute the onset of their illness to a combination of work and non-work stressors [18]. Self-rated ED is based on the Swedish diagnostic criteria for ED, but it is not a medical diagnosis. It is rather a measure of a stressed individual’s recognition of their condition and perception of its severity and effects on their well-being. The s-ED scale is a screening instrument, developed for assessment of ED, and the s-ED scale strives to be compliant with the diagnostic criteria for ED [19]. Previous studies have found s-ED prevalence rates of 7.8% to 21% among working people [19–22]. This study will focus on s-ED among municipal employees in rural northern Sweden.

Excessive workload and psychologically stressful work are associated with working in the municipal sector in Sweden [23], Norway [24], and Finland [25]. Research on the Swedish municipal sector has shown that high workload is associated with mental health problems, especially in those who have low social support [26]. A study among municipal school principals showed that almost one in three reported signs of possible ED [27]. Research has described shortages in work health promotion, where municipal organizations focus on individual health, rather than factors related to the work environment, and also a lack of follow-up after e.g. the annual employee survey [28]. Municipal employees have the highest sick-leave rates in the country, most commonly for stress-related disorders and with a twofold prevalence in women over men [29]. Among municipal employees, where the major occupational groups in the municipal sector are social workers, preschool and school staff, and elderly care employees, lack of recovery time is thought to be an important link between working conditions, ill health, and sick leave [30]. The importance of having time and energy for both private life and work have been described as essential among home help service nurses, and shift work and part time work as two resources contributing to flexibility and a prerequisite to better work-life balance [31]. General work experience such as joy, a good atmosphere, feedback and meaningfulness in work has found to be the strongest predictor of health among municipal health care staff [32], and poor health has been associated with high rates of long-term sick leave, and found in municipalities with a population decline [33].

A population decline is often found in Sweden’s rural municipalities, where young and highly educated people move, leaving the population older and low educated. Rural municipalities are often small in population but large in land area, and located far away from the growth regions [34]. Research in the Nordic countries, Denmark, Finland, and Norway report poorer self-reported health and higher prevalence of obesity, and physical inactivity in rural areas compared to urban areas; however, the opposite pattern was reported in Sweden [35, 36]. Mortality has been reported higher in municipalities with low population density in Sweden, Norway and Finland [37]. Research in northern Sweden has shown higher level of cardiovascular risk factors as obesity, high cholesterol and sedentary lifestyle when living in rural areas, compared to urban areas [38], but lower risk of mental disorder sick leave in sparsely populated areas than urban areas [39]. Recent results from a study conducted in northern Sweden’s rural municipalities showed an s-ED prevalence of 21.5% among rural municipal employees [40].

The results mentioned above indicate employees’ health and well-being depend on a balance of positive and negative organizational and psychosocial environmental work factors, that is demands and resources. In Sweden, a large proportion of sick leave can be linked to occupational stress, and its association with the organizational and psychosocial work environment has increased over time. Exhaustion disorder (ED) is a common cause of sick leave, which for this diagnosis often exceeds 6 months. Job insecurity, low influence of work-related decisions, high effort for low reward, and lack of support are important organizational and psychosocial factors related to ED. Working in the municipal sector and in the rural context could both contribute to health problems, poorer organizational and psychosocial work environments, and higher sick-leave rates. Little is known about s-ED among working municipal employees in rural northern Sweden. To our knowledge, no research has focused on associations between the organizational and psychosocial work environment and s-ED. This cross-sectional study can therefore contribute to increased knowledge and inform future interventions to promote healthy workplaces, increase well-being, and prevent long-term sick leave due to ED.

### 1.1 Aim

The aim of this study was to explore organizational and psychosocial environmental work factors among municipal employees with or without self-rated exhaustion disorder (s-ED) in rural northern Sweden.

### 1.2 Research questions

What organizational and psychosocial environmental work factors are associated with municipal employees with s-ED, and what factors are associated with employees without s-ED?

Are there between-group differences in organizational and psychosocial environmental work factors among employees with or without s-ED?

Are there differences in organizational and psychosocial environmental work factors in the two groups compared to national reference values?

## 2 Methods

### 2.1 Study design and procedure

We performed this cross-sectional study in 2018 in two rural municipalities in northern Sweden, using a web-based questionnaire to collect data from March to June. All municipal employees in the two municipalities received a link to the questionnaire by e-mail. For employees without a known e-mail address, data were collected through a paper-based questionnaire. Three reminders were sent to non-responders by e-mail or paper mail as appropriate. The questionnaire asked for background variables and included instruments measuring participants’ organizational and psychosocial work environment and s-ED. This study was performed on behalf of a coordination association in the area, selecting the two municipalities and municipal employees included in this study.

### 2.2 Settings and subjects

The Swedish Board of Agriculture [41] defines rural areas in terms of population density and proximity to a city. There could, however, be large between-country differences in definitions of rural areas, despite apparent similarities among the defining factors used to describe rural areas in research [42]. In the present study, municipality 1 (1600 square kilometres, ∼618 square miles) has about 3100 inhabitants, and municipality 2 (5500 square kilometres, ∼2125 square miles) has about 12 200 [43]. Of 2077 municipal employees asked to participate in the study, 1093 (52.6%) answered the questionnaire. Three persons could not be categorized as s-ED or non–s-ED because of missing internal values, thus 1090 persons completed the s-ED scale. The dataset in this study and demographic characteristics of the employees of the two municipalities have been previously reported [40]. Regarding professions in Table 1, ‘nursing staff’ and, ‘educational staff’ refers to having a human service profession in each sector. ‘Office staff’ refers to employees with a desk or administrative work, managers excluded. ‘Managers’ refers to having a leading role and being responsible for subordinates. ‘Non-office staff’ refers to other employees with a practical work, e.g. cleaner, janitor, construction worker and cashier.

**Table 1:** Characteristics of participants with or without s-ED (*n* = 1090)

	s-ED	non–s-ED	*p* value	Effect size
	234 (21.5)	856 (78.5)
Municipality
Municipality 1	69 (29.5)	195 (22.8)	0.034	*φ*= –0.064
Municipality 2	165 (70.5)	661 (77.2)
Sex
Male (%)^*^	35 (15.0)	224 (26.2)	<0.001	*φ*= 0.108
Female (%)^*^	199 (85.0)	632 (73.8)
Age (range 19–67)
Mean years±SD^*^	42.4±12.3	45.8±11.9	<0.001	*d* = 0.281
Employment
Work full time (%)	154 (65.8)	631 (73.7)	0.015	*φ*= 0.074
Work part time (%)	80 (34.2)	223 (26.1)
Time at current workplace (range 0–42)
Mean years±SD	7.8±7.6	9.0±8.9	0.057	*d* = 0.145
Time as municipal employee (range 0–45)
Mean years±SD	13.3±10.3	15.7±11.3	0.003	*d* = 0.206
Long-term sick leave
No (%)	199 (85.0)	825 (96.4)	<0.001	*φ*= 0.195
Yes (%)	35 (15.0)	31 (3.6)
Children living at home
Yes (%)	108 (46.2)	414 (48.4)	0.555	*φ*= –0.018
No (%)	125 (53.4)	439 (51.3)
Working schedule
Day/evening (%)	178 (76.1)	702 (82.0)	0.041	*φ*= 0.062
Night (%)	56 (23.9)	154 (18.0)
Marital status
Living with a partner (%)	173 (73.9)	685 (76.9)	0.050	V = 0.074
Living apart together (%)	12 (5.1)	22 (2.6)
Single (%)	49 (20.9)	149 (17.4)
Education
Compulsory school (%)	6 (2.6)	35 (4.1)	0.339	V = 0.045
Upper secondary school (%)	122 (52.1)	409 (47.8)
University (%)	106 (45.3)	412 (48.1)
Living
Urban area (%)	178 (76.1)	628 (73.4)	0.404	*φ*= –0.025
Rural area (%)	56 (23.9)	228 (26.6)
Home
House (%)	149 (63.7)	619 (72.3)	0.024	V = 0.084
Apartment (%)	84 (35.9)	229 (26.8)
Other (%)	1 (0.4)	7 (0.82)
Profession
Nursing staff (%)	96 (41.0)	280 (32.7)	0.001	V = 0.133
Educational staff (%)	107 (45.7)	349 (40.8)
Managers (%)	12 (5.1)	65 (7.6)
Office staff (%)	11 (4.7)	107 (12.5)
Non-office staff (%)	8 (3.4)	53 (6.2)

^*^These figures have earlier been published [40].

### 2.3 Instruments

#### 2.3.1 S-ED scale

The s-ED scale [19] was used to assess municipal employees’ self-rated levels of exhaustion. The scale is based on the Swedish diagnostic criteria for ED. Being classified as having s-ED requires a *Yes* statement to questions 1, 2, and 4 and affirmation of at least four of the six symptoms in question 3. An individual must 1) feel physically and/or mentally exhausted for more than two weeks; 2) consider this exhaustion to be caused by long-term stress exposure (6 months or more); 3) experience symptoms for the last 2 weeks such as concentration or memory problems, markedly reduced capacity to tolerate demands or to work under time pressure, emotional instability or irritability, sleeping problems, physical weakness or being more easily fatigued, physical symptoms such as muscular pain, chest pain, palpations, gastrointestinal problems, vertigo, or increased sensitivity to sounds; 4) the complaints above have markedly decreased well-being and/or functional capacity. The s-ED scale distinguishes between light/moderate and pronounced s-ED in question number four with the response options ‘yes, to a great extent’, ‘yes, somewhat’ or ‘no, not at all’. The instrument has been validated in a study of health and medical staff in Sweden, which showed good construct validity [19].

#### 2.3.2 Organizational and psychosocial work environment

The Modern Work Life Questionnaire (MWQ) [44] measures organizational and psychosocial work environmental factors (e.g., demands, control, and support), and is based on questions that has been found to be both theoretically and empirically important. During the initial validation of the questionnaire, principal component analysis (PCA) was used to analyses the construct validity. The MWQ has been judged as valid and sufficiently reliably for mapping the organizational and psychosocial work environment [44, 45]. The Modern Work Life Questionnaire contains 127 questions (i.e. items) about organizational and psychosocial work environment and health. The 127 items in the MWQ form a total of 77 factors, and every factor consists of one or several items. Of the total of 77 factors, 32 factors were included in this study, focusing on the organizational and psychosocial environmental work factors considered most important in relation to ED based on theory and empirical data, such as demands, social support, resources, and conflicts. One example is the factor quantitative demands (QD), which consists of three questions ‘Does your job demand that you work very fast?’ ‘Does your job demand that you work very hard?’ ‘Does your work demand too much effort?’ (‘yes, often’, ‘yes, sometimes’, ‘no, rarely’, or ‘no, never’). The number of response options on different questions range from two to seven. The time period for the questions varies from latest week, 3 months to 2 years (e.g., organizational changes), while some questions do not specify a time period [44, 45]. The results from an individual workplace can be compared with reference values from a representative large sample of the working population in Sweden reported in the national Swedish Longitudinal Occupational Survey of Health (SLOSH). The SLOSH is a longitudinal survey with focus on the association between organization, work environment, and health. The national Swedish Longitudinal Occupational Survey of Health (SLOSH) is based on a representative large sample of the working population in Sweden, from which national reference values has been obtained. These reverence values of SLOSH are representative mean values and proportions for the working population in Sweden, and the same corresponding values for other sub-groups can be calculated in contrast to the working population [46, 47]. The scoring directions of all factors are clarified in Table 2.

**Table 2:** Differences in factors in organizational and psychosocial work environments among municipal employees with and without s-ED (*n* = 1090)

	s-ED	non–s-ED	Reference value	Effect size
	234 (21.5)	856 (78.5)
Quantitative demands (QD)^*^(range 1–4), mean±SD (95% CI)	1.70±0.53 (1.63–1.77)	2.08±0.57 (2.04–2.12)	2.10	*d* = 0.678
Emotional demands (EmD)^*^(range 1–4), mean±SD (95% CI)	1.44±0.49 (1.38–1.51)	1.78±0.68 (1.73–1.82)	2.28	*d* = 0.574
Intellectual demands (ID)^*^(range 1–4), mean±SD (95% CI)	1.34±0.45 (1.28–1.40)	1.51±0.51 (1.48–1.55)	1.54	*d* = 0.374
Social competence (SC)^*^(range 1–4), mean±SD (95% CI)	1.14±0.38 (1.09–1.19)	1.25±0.50 (1.21–1.28)	1.48	*d* = 0.248
IT demands (ITD)^*^(range 1–5), mean±SD (95% CI)	2.90±0.95 (2.78–3.02)	3.21±0.80 (3.15–3.26)	2.87	*d* = 0.353
Physical demands (PD)^*^(range 1–6), mean±SD (95% CI)	4.19±1.75 (3.96–4.42)	4.74±1.44 (4.65–4.84)	4.77	*d* = 0.343
Social support (SOC)^**^(range 1–4), mean±SD (95% CI)	1.97±0.73 (1.88–2.06)	1.60±0.55 (1.56–1.63)	1.84	*d* = 0.572
Possibilities to influence (PI)^**^(range 1–4), mean±SD (95% CI)	2.07±0.73 (1.98–2.17)	1.77±0.64 (1.72–1.81)	1.80	*d* = 0.437
Resources (RES)^**^(range 1–4), mean±SD (95% CI)	2.10±0.68 (2.01–2.19)	1.68±0.60 (1.64–1.72)	1.5	*d* = 0.655
Time^**^(range 1–4), mean±SD (95% CI)	2.51±0.75 (2.41–2.60)	1.92±0.70 (1.87–1.97)	2.1	*d* = 0.813
Knowledge^**^ (KNOW)(range 1–5), mean±SD (95% CI)	3.00±0.85 (2.89–3.11)	3.05±0.73 (3.00–3.09)	2.93	*d* = 0.063
Working autonomy (WA)^**^(range 1–4), mean±SD (95% CI)	2.43±0.76 (2.33–2.53)	2.07±0.68 (2.02–2.11)	2.35	*d* = 0.499
Opportunities to influence working hours (OIWH)^*^(range 1–6), mean±SD (95% CI)	2.41±1.16 (2.26–2.56)	2.90±1.22 (2.82–2.99)	2.88	*d* = 0.412
Participation in decisions (PID)^**^(range 1–5), mean±SD (95% CI)	3.04±0.84 (2.94–3.15)	2.77±0.84 (2.71–2.82)	2.56	*d* = 0.321
Workplace democracy (WD)^**^(range 1–3), mean±SD (95% CI)	2.08±0.55 (2.01–2.15)	1.78±0.49 (1.75–1.83)	2.02	*d* = 0.576
Manifested freedom of expression (MFE)^**^ (range 1–4), mean±SD (95% CI)	1.93±0.85 (1.82–2.04)	1.72±0.69 (1.67–1.76)	1.94	*d* = 0.271
Belonging (BEL)^**^(range 1–5), mean±SD (95% CI)	2.05±0.95 (1.93–2.17)	1.70±0.68 (1.65–1.74)	1.82	*d* = 0.424
Human beings versus profitability (HUP)^**^(range 1–4), mean±SD (95% CI)	2.53±0.84 (2.43–2.64)	2.11±0.75 (2.06–2.16)	2.30	*d* = 0.527
Values (VAL)^**^(range 1–5), mean±SD (95% CI)	2.36±0.89 (2.24–2.47)	1.98±0.69 (1.93–2.03)	2.11	*d* = 0.477
Salary (SAL)^**^(range 1–4), mean±SD (95% CI)	3.04±0.81 (2.94–3.15)	2.59±0.80 (2.54–2.64)	2.46	*d* = 0.559
Confidence in management (CM)^**^ (range 1–4), mean±SD (95% CI)	2.61±0.92 (2.49–2.73)	2.11±0.78 (2.06–2.16)	2.38	*d* = 0.586
Relation to immediate manager (RIM)^**^ (range 1–4), mean±SD (95% CI)	2.21±0.88 (2.09–2.32)	1.86±0.68 (1.81–1.90)	2.19	*d* = 0.445
Coordination (COR)^**^(range 1–4), mean±SD (95% CI)	2.38±0.99 (2.26–2.51)	1.99±0.78 (1.93–2.04)	2.39	*d* = 0.438
Presence of immediate manager (PM)^**^ (range 1–4), mean±SD (95% CI)	2.51±0.98 (2.39–2.64)	2.28±0.93 (2.22–2.34)	1.63	*d* = 0.241
Organizational structure (OS)^**^(range 1–4), mean±SD (95% CI)	2.30±0.70 (2.21–2.39)	1.91±0.52 (1.88–1.95)	2.00	*d* = 0.632
Organizational barriers (OB)^*^(range 1–4), mean±SD (95% CI)	2.43±0.85 (2.32–2.54)	2.74±0.75 (2.69–2.79)	2.78	*d* = 0.387
Downsizing and relocation (DAR)^*^ (range 1–5), mean±SD (95% CI)	3.22±0.95 (3.10–3.35)	3.64±0.98 (3.57–3.71)	3.94	*d* = 0.435
Reorganization (REORG)^**^(range 1–4), mean±SD (95% CI)	2.21±0.92 (2.09–2.33)	1.89±0.82 (1.83–1.94)	1.71	*d* = 0.367
Conflicts with managers (CWM) % (n) (95% CI)	26.1% (61) (20.40–31.73%)	10.3% (88) (8.24–12.32)	18.3	*φ*= –0.189
Conflicts with co-workers (CWC) % (n) (95% CI)	35.5% (83) (29.29–41.65)	19.2% (164) (16.52–21.80)	20.3	*φ*= –0.160
Conflicts with others (CWO) % (n) (95% CI)	36.8% (86) (30.53–42.98)	23.5% (201) (20.64–26.33)	24.4	*φ*= –0.124
Violence or threat of violence (VTV) % (n) (95% CI)	32.1% (75) (26.02–38.07)	23.1% (198) (20.30–25.96)	16.1	*φ*= 0.085

^*^Lower values indicate more negative experienced factors of the organizational and social environment. ^**^Lower values indicate more positive experienced factors of the organizational and social environment.

### 2.4 Statistical analyses

Statistical analyses were performed using SPSS version 25.0 [48], and programming language R (R version 3.5.1, 2018-07-02). Cronbach’s alphas where calculated for all factors (subscales) under study when applicable. The factor intellectual demands (ID) had a Cronbach’s alpha of 0.51 and the factor Downsizing and relocation (DR) had a Cronbach’s alpha of 0.55. The Cronbach’s alphas regarding the other factors ranged from 0.71 (Time) to 0.91 (IT demands, ITD). Descriptive statistics are presented as mean scores, standard deviations (SDs), and frequency distributions when applicable. Group comparisons regarding the characteristics of participants were made using *t*-test and chi-square depending on the characteristics of the variable (Table 2). Group comparisons were made using 95% confidence intervals to compare means and proportions. The 95% confidence intervals (CI) were also used to make comparisons to the reference values. The factor means of the Modern Work Life Questionnaire were calculated according to the instructions from one of the responsible for the questionnaire (J. Gustafsson, personal communication by e-mail, December 5, 2017). That is, individual means to each factor was first calculated by summarizing all items to each factor in the MWQ, and then divide with the number of items related to the factor. Secondly, mean values were calculated based on these individual means as instructed. In addition to statistical significance, effect sizes were analysed using Cohen’s *d* (*d*), phi coefficient (*φ*), and Cramer’s *V* (*V*). Cohen’s criteria consider the effect sizes for *d* value of 0.2 as representing a small effect, a value of 0.5 as representing a medium effect, and a value of 0.8 as representing a large effect. Effects of *φ* values of 0.10 are considered small, 0.30 moderate, and 0.5 large [49]. The criteria for the effect size measured by *V* varies depending on the number of categories; the criteria described by Pallant was used [50].

**Fig. 1:**
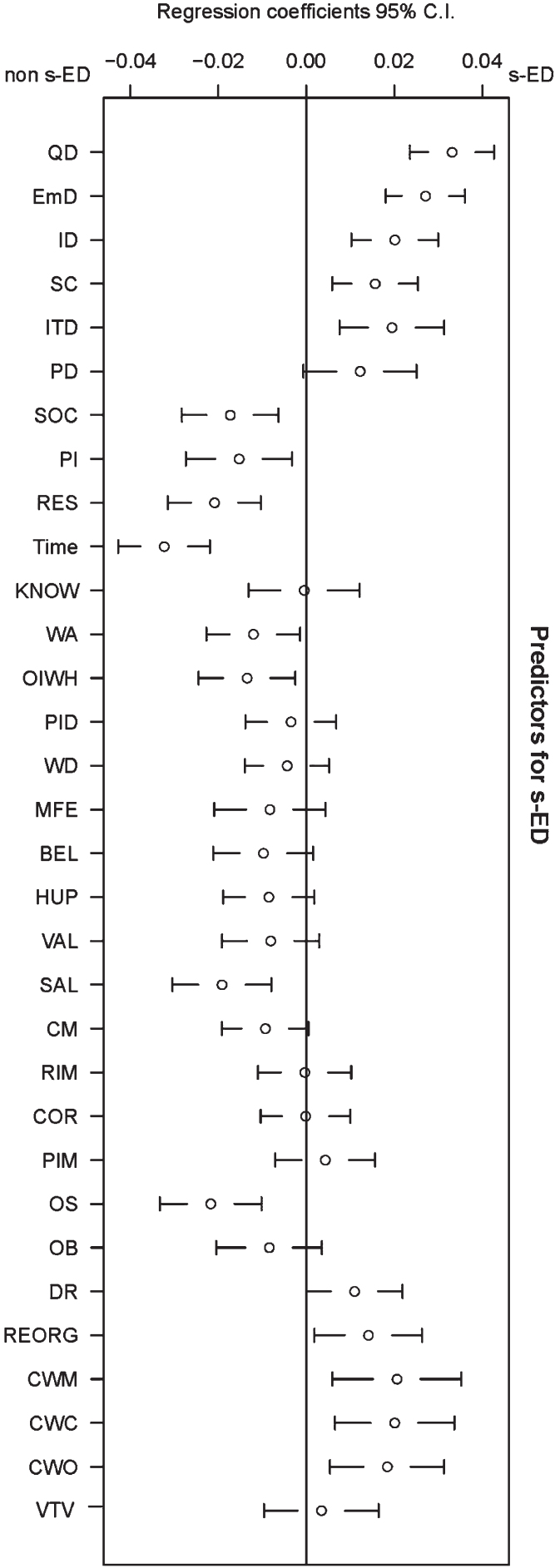
Plots of jackknife 95% confidence intervals around the regression coefficients from PLSR for organizational and social environment factors in the MWQ. Important factors for belonging to the non–s-ED group are presented to the left. Important factors for belonging to the s-ED group are presented to the right. The abbreviations are explained in Table 3.

Partial least square regression (PLSR) was used to assess the most important predictive factor(s) in municipal employees’ belonging to the with s-ED or without s-ED group. Factor scores were received by calculating the total scores for each of the 32 factors (i.e. the sum of all scores from all the items within each factor). Before performing the PLSR, some factors were reversed in order to facilitate interpretation of the results. The number of components in the PLSR models was selected by examining validation plots for mean square error of prediction (MSEP), root mean square error of prediction (RMSEP), coefficient of multiple determination (R^2^) and by leave-one-out cross-validation as recommended by Mevik and Cederkvist [51]. Two components were regarded as sufficient. To facilitate the interpretation of the results of the PLSR model, a figure was produced showing the predictive patterns of the factors in the MWQ in relation to the response variables (s-ED/non-s-ED). In other words, a figure containing each regression coefficient for each factor with jackknife 95% confidence intervals (CIs) surrounding the regression coefficients (Fig. 1).

**Table 3:** Description of the 32 organizational and psychosocial environmental work factors in the PLSR model

Factor	Abbreviation	Summary
Quantitative demands	QD	Working too fast, working too hard
Emotional demands	EmD	Understanding others’ situations, being exposed to difficult emotional situations at work
Intellectual demands	ID	Constantly learning new things, problem solving
Social competence	SC	Work requiring great social skills
IT demands	ITD	Being stressed by too many phone calls and e-mails, being interrupted, give quick replies
Physical demands	PD	Physically heavy work
Social support	SOC	Team cohesion at work, support from co-workers
Possibilities to influence	PI	Freedom to decide what to prioritize in work and how the work should be performed
Resources	RES	Enough staff, economic resources, and equipment
Time	Time	Enough time for work and for reflection
Knowledge	KNOW	Enough work-related knowledge and skills
Working autonomy	WA	Freedom at work to decide what to do
Opportunities to influence working hours	OIWH	Opportunities to affect working hours (start, stop, and break times and days at work)
Participation in decisions	PID	Involvement in decision making in the immediate workplace and overall organization
Workplace democracy	WD	Feeling opinions matter at work, having enough information before important decisions, shared participation in discussions
Manifested freedom of expression	MFE	Ability to express to the manager thoughts, feelings, and wishes about work
Belonging	BEL	Sense of belonging in the workplace
Human beings versus profitability	HUP	Caring for human beings as much as profitability
Values	VAL	Match between workplace and personal values
Salary	SAL	Satisfaction with salary
Confidence in management	CM	Confidence in workplace management
Relation to immediate manager	RIM	Relationship with immediate manager (does the manager listen, and give confirmation?)
Coordination	COR	Ability of manager to coordinate overall work operations
Presence of immediate manager	PIM	Presence of immediate manager in the workplace
Organizational structure	OS	Clear working rules and roles
Organizational barriers	OB	Obstacles to work created by organizational structures
Downsizing and relocation	DR	Downsizing and relocations in the workplace
Reorganization	REORG	Any workplace reorganization in the past 2 years
Conflicts with managers	CWM	Conflicts with managers in the past 2 years
Conflicts with co-workers	CWC	Conflicts with co-workers in the past 2 years
Conflicts with others	CWO	Conflicts with others in the past 2 years
Violence or threat of violence	VTV	Exposure to violence or threats of violence at work

### 2.5 Ethics

This study was approved by the Swedish Ethical Review Authority, Dnr 2017/495-31. Before we emailed the link to the questionnaire to potential participants, we informed them in an introductory letter about the voluntary nature of their participation and our assumption that their completion of the questionnaire would signify their consent to participate. Thereby, informed consent was obtained by all participants.

## 3 Results

There were a total of 1093 municipal employees in the two rural areas of northern Sweden. Of these, 261 were men (23.9%) and 831 women (76.2%). The mean age was 45.1 years, and nearly half of the municipal employees had a university education (47.5%). The majority lived in Municipality 2 (75.8%), and the mean time as municipal employee was 15.2 years. Most employees worked in nursing (34.6%) or education (41.8%). Table 1 shows comparisons of background characteristics between municipal employees with or without s-ED. The s-ED group had a significantly lower mean age (mean = 42.4) compared to the non–s-ED group (mean = 45.8; *p* < 0.001; d = 0.281). Within the s-ED group, there was significantly higher proportion of women (85.0%) than men (15%; *p* < 0.001; *φ*= 0.108). A significantly higher proportion of employees in the non–s-ED group worked full time (73.7%), compared to the s-ED group (65.8%; *p* = 0.015; *φ*= 0.074).

Results of the univariate analyses are presented in Table 2. There were significant differences between the two groups regarding all factors except the knowledge factor. The s-ED group reported significantly higher quantitative (m = 1.70, CI: 1.63–1.77) and emotional demands (m = 1.44, CI: 1.38–1.51) than the non–s-ED group (m = 2.08, CI: 2.04–2.12; m = 1.78, CI: 1.73–1.82, d = 0.678). Social support from workplace colleagues was significantly higher among employees in the non–s-ED group (mean = 1.60, CI: 1.56–1.63) than in the s-ED group (mean = 1.97, CI: 1.88–2.06, d = 0.572). The employees in the non–s-ED group assessed having significantly more resources (m = 1.68, CI: 1.64–1.72) (i.e. enough staff, economic resources, and equipment) than the s-ED group (m = 2.10, CI: 2.01–2.19, d = 0.655). The employees in the non–s-ED group assessed having significantly more time for work and reflection (mean = 1.92, CI: 1.87–1.97) than the s-ED group (mean = 2.51, CI: 2.41–2.60, d = 0.813). Univariate results also showed that a significantly larger proportion of employees in the s-ED group reported conflicts with managers (21%, CI: 20.40–31.73) and co-workers (35%, CI: 29.29–41.65) compared to the non–s-ED group (managers: 10.3%, CI: 8.24–12.32; co-workers: 19.2%, CI: 16.52–21.80). The differences were small (managers: *φ*= –.189, co-workers: *φ*= –.160).

Compared to the national reference values (NRV) both the s-ED and the non–s-ED groups in this study assessed significantly higher emotional demands (NRV EmD = 2.28) and fewer resources (NRV RES = 1.5) as there were no overlapping CI; s (see above). Both groups also assessed being more frequently exposed to violence or threats of violence compared to the NRV (16.1%). In the S-ED group 32.1% of the employees assessed being exposed to violence or threats (CI: 26.02–38.07) and the corresponding figures for the employees in the non–s-ED group was (23.1%, CI: 20.30–25.96).

Compared to the national reference values the s-ED group reported less time for work (NRV Time = 2.1) and the non–s-ED group reported more time for work, as there were no overlapping CI; s. Compared to the national reference values the s-ED group assessed significantly higher quantitative demands (NRV QD = 2.10). Compared to the national reference values the s-ED group reported less social support (NRV SOC = 1.84), and the non–s-ED group reported more perceived social support, as there were no overlapping CI; s (see above). The s-ED group reported higher proportional exposure to conflicts with managers (26.1%, CI: 20.40–31.73%) and co-workers (35.5%, CI: 29.29–41.65) compared to the NRV; s (CWM = 18.3%, CWC = 20.3%).

The organizational and psychosocial environmental work factors in the PLSR model (Fig. 1) explained 21.0% of the variance in the response variable (s-ED/non–s-ED). Important factors of belonging to the s-ED group were different types of demands: quantitative, emotional, intellectual, and IT demands. Quantitative demands (having to work too fast or too hard) and emotional demands (understanding and being exposed to others’ often difficult emotional situations) were the most important types of demands belonging to the s-ED group. Other important factors were low social competence, reorganization, conflicts with managers, co-workers, and with others. Important factors for belonging to the non–s-ED group were social support, possibilities to influence, resources, time, work autonomy, opportunities to influence working hours, salary and organizational structure. All 32 factors (organizational and psychosocial work environmental factors) in the PLSR are summarized in Table 3. Regression coefficients, standard errors and *p*-values from jackknife *t*-tests are described in Table 4.

**Table 4:** Regression coefficients, standard errors and *p*-values from jackknife *t*-tests

Factor	Regression coefficient	Standard error	*p*-value
QD	0.0331	0.0049	1.7e-11
EmD	0.0271	0.0046	4.7e-09
ID	0.0201	0.0050	6.9e-05
SC	0.0157	0.0049	0.0017
ITD	0.0195	0.0061	0.0014
PD	0.0122	0.0066	0.0627
SC	–0.0173	0.0056	0.0021
PI	–0.0153	0.0062	0.0132
RES	–0.0209	0.0054	0.0001
Time	–0.0323	0.0053	1.6e-09
KNOW	–0.0005	0.0064	0.9399
WA	–0.0121	0.0054	0.0269
OIWH	–0.0135	0.0056	0.0164
PID	–0.0035	0.0053	0.5055
WD	–0.0044	0.0049	0.3680
MFE	–0.0083	0.0065	0.2001
BEL	–0.0098	0.0058	0.0926
HUP	–0.0085	0.0053	0.1063
VAL	–0.0081	0.0056	0.1516
SAL	–0.0192	0.0057	0.0008
CM	–0.0093	0.0050	0.0630
RIM	–0.0004	0.0054	0.9461
COR	–0.0002	0.0052	0.9740
PIM	0.0043	0.0058	0.4573
OS	–0.0218	0.0059	0.0002
OB	–0.0084	0.0061	0.1695
DR	0.0109	0.0056	0.0499
REORG	0.0141	0.0062	0.0239
CWM	0.0206	0.0075	0.0060
CWC	0.0201	0.0069	0.0038
CWO	0.0184	0.0066	0.0055
VTV	0.0034	0.0067	0.6055

## 4 Discussion

This cross-sectional study explored organizational and psychosocial environmental work factors among municipal employees with or without self-rated exhaustion disorder (s-ED) in rural northern Sweden. The results showed that there were significant differences between the s-ED and the non–s-ED group in all but one of the organizational and psychosocial environmental work factors. Various demands were associated with the s-ED group, and resources were associated with the non–s-ED group. Both groups assessed assess higher emotional demands and less resources compared to the national reference values.

An overall understanding is that the results from this study conform with the JD-R model [5], that demands can give rise to health impairment and burnout, while job resources buffer the health-impairing impact of demands and burnout model [7]. The overall results also point toward that access to various resources and lower demands can be protective factors against s-ED. One reflection is that it can be important for those working with improving occupational health in municipalities to be aware of these potentially protecting factors against ED. Such knowledge can be used in order to promote well-being among municipal employees. A recent review has described that ED is highly unexplored internationally, and that the medical diagnosis of exhaustion disorder has not yet been accepted into international versions of the ICD [52]. This means that straight forward comparisons to previous research of ED is limited. However, research has shown that ED overlaps with the concept of clinical burnout [13], consequently it seems reasonable to make comparisons to burnout and other stress-related disorders.

Both univariate and multivariate results showed that employees in the s-ED group reported higher quantitative and emotional demands than those in the non–s-ED group. These results are in line with previous results from several reviews concluding that quantitative and emotional demands are associated with increased emotional exhaustion and stress-related disorders [53, 54]. Multivariate results showed that the factor quantitative demands had the strongest association to the group of municipally employees with s-ED out of all factors in the present study. This is in accordance with a previous longitudinal study, that showed quantitative demands to have the largest impact on perceived effort (stress) compared to all the other job demands. Perceived effort in turn significantly increases burnout among nurses [55]. This can be problematic as results have shown that burnout is a significant factor of several negative physical and psychological consequences on workers well-being and health [56]. Burnout has also been shown to be associated with an increased intention to leave the nursing profession [55], decreased quality of care [57], negatively affect children’s academic skills if teachers suffer from feelings of burnout [58]. The results from a longitudinal study among working employees in Sweden showed that high demands were associated with greater risk of burnout, regardless of whether employees were working in a supportive or unsupportive work environment [59]. A cross-sectional and longitudinal study found that increased emotional demands were associated with increased exhaustion among Danish public service employees. Furthermore, high levels of quantitative demands were found to increase the effect of emotional demands on exhaustion [60]. Thus, in order to counteract such negative effects, it seems important to decrease levels of s-ED among municipal employees by organizing the workplaces in such a way that demands are decreased. It can be fruitful to try and specifically reduce quantitative demands. This may improve the well-being and health of those municipally employees at risk of becoming sick of ED and by extension improve the quality of their work in schools and in residential care of older people.

Univariate and multivariate results in the present study show that employees in the s-ED group reported more conflicts with managers and co-workers than those in the non–s-ED group. Between-group differences in exposure to conflict with managers and co-workers were small. A previous cross-sectional study among Finnish municipal employees showed that psychological harassment, workplace bullying, and injustice in the workplace were associated with exhaustion [61]. Workplace conflicts have also been reported in Sweden as important contributors to stress-related illness among people on sick leave for ED [18]. The present study also showed that both groups assessed being exposed to more violence or threats of violence compared to the NRV;s. Among municipal employees in Sweden, 27% (13% of the total labour market) have reported being exposed to violence or threat of violence [23]. A previous cross-sectional study showed that one third of public sector employees in Sweden were exposed to violence or threats of violence showed a relationship between work-related violence and poorer health [62]. Other research among human service sector occupations has found psychosocial work environment factors e.g. high quantitative and emotional demands, low organizational justice, and low level of influence over own work-situation to be were associated with work-related threats. High emotional demands, low quality of leadership and low support from nearest supervisor were some factors associated with workplace violence [63]. The results of the present study indicate how important it is for employers to deal with adverse organizational climates to limit risk factors for employees’ developing ED and consequently taking long-term sick leave.

Both univariate and multivariate results showed that the non–s-ED group reported having more social support, resources and time for work than the s-ED group. The between-group differences in mean scores for social support and resources were moderate, but large for the time factor. In addition, the results also showed that S-ED group reported less time for work and social support in contrast to the NRV;s. Furthermore, the non-s-ED group assessed more time for work and social support than the NRV;s. Previous reviews have concluded that employees who felt unsupported in the workplace developed more symptoms of ED [17, 64], and that good support at work can protect against ED [53]. Results of a previous cross-sectional study have shown an association between low social support and exhaustion among working employees in Sweden [65]. Findings from a qualitative study has found that people with ED on long-term sick leave consider the support of supervisors and co-workers important to their chances of regaining their ability to work [66]. Qualitative research among school principals has also shown the importance of social support from both managers and co-workers for occupational well-being [25], and the availability of workplace resources has been found to improve both employee well-being and work performance [67]. Time pressures, overtime requirements, lack of time for reflection at work or recovery after (all described as common in human service occupations), and lack of resources at work to meet these demands can upset employees’ work/life balance [68], and cause stress and exhaustion. Using the JD-R model, job resources (e.g., social support, autonomy) were negatively related to burnout [69], and positively related to employee well-being in Norway [70]. It seems important to focus on organizational and psychosocial protective factors: that is, to organize work such that employees have enough time both to perform and to reflect upon their work tasks. It is also important to provide sufficient economic and staff resources, as well as access to a supportive and present manager, to prevent s-ED in the vulnerable rural municipal services sector.

Interestingly, compared to the NRV;s both the s-ED and the non–s-ED groups in this study assessed higher emotional demands and fewer resources. Results also showed that s-ED group assessed higher quantitative demands than the NRV;s. Possible explanations for these results may partly be the population decline facing many rural areas both in Europe [71] and Sweden, and its consequences. Some financial and organizational consequences as centralise the care of the elderly, school closure, extensive budget cuts, shortage in the workforce in certain sectors, and difficulties recruiting for example certified nurses and teachers, which could in turn put added pressure on municipal employees in school and elderly care who are asked to do more with less. It may also be difficult change employers in a rural municipality [34]. Furthermore, employees in rural areas have been reported to have a different work situation than those in urban areas [72]. However, some of the challenges described above has also been reported to be present in municipalities in urban areas with a population increase [73, 74], and it is known that working in municipal sector is associated with stressful work environments [23]. Consequently, it is possible that the high demands and lack of resources is a consequence of the challenges facing municipalities, regardless if they are located in rural areas or not. Future studies are needed in order to explore this issue.

### 4.1 Methodological discussion

It has been suggested that Cronbach’s alpha should be somewhere between 0.7–0.95 [75]. Two factors had Cronbach’s alpha values below the recommended interval: intellectual demands (*α*= 0.51) and downsizing and relocation (*α*= 0.55). However, it should be noted that these two factors only contain two items each and the low values can be an indication that some additional items are needed. Cronbach’s alpha values regarding the other factors where within the recommended interval and points toward satisfactory scale reliability. It should be noted that the response rate in this study of 52.6% is a cause of concern since it could indicate that non-response bias can be present. There were differences between responders and non-responders regarding profession and municipal belonging, but the effect sizes were small. Nursing staff, and non-office staff had the highest number of non-responders (58.4% and 61.1%). This was partly expected since previous research among healthcare professions has shown a similar response rate (56%) in postal surveys [76]. Research has shown that non-respondents had 20–30% higher sick-leave rate compared to respondents [77]. It is possible that the non-responders working as nursing staff, and non-office staff could suffer from more ill-health compared to other professions, and that the prevalence of s-ED was slightly underestimated in the current study. However, the results point towards the opposite. There was a significantly higher proportion of responders in municipal 1, and a significantly larger proportion of municipal employees with s-ED. This indicates that the potential problem of underestimating s-ED might be a limited problem. The response rate in this study is slightly higher than could be expected for web-based studies [78]. The sample can be regarded as nationally representative in terms of sex and age [79]. A limitation of this study is the cross-sectional design as no insights about causality can be provided. In addition, it should be noted that the NRV;s is based on a representative large sample of the working population in Sweden while the current study is based on a selected sample solely consisting of municipal employees in rural northern Sweden. Consequently, it is not possible to make a direct comparison and use proposed reference values based on the NRV data. It also should be noted that the current study may be at risk of common method variance (CMV) [80]. However, research has found that a relatively high level of CMV must be present to bias a true correlation between variables [81]. Many regression techniques perform poorly with large number of variables and when there are high co-variance values as the case in the current study. However, a strength with PLSR is that it works well with small samples and many variables and is robust with inadequacies such as high co-variance values [82].

## 5 Conclusion

The results from this study are relevant to a better understanding what organizational and psychosocial work environmental factors the employer need to pay extra attention to, since this study provides increased knowledge of various work factors associated with s-ED and non-s-ED. This study shows that municipal employees with s-ED rated their total organizational and psychosocial work environment as poorer than did employees in the non–s-ED group, and that municipal employees in rural northern Sweden assess higher emotional demands and less resources compared to the national reference values. These are new insights, since such comparisons have not been made previously to our knowledge. Increased workplace awareness is important in identifying employees who experience adverse working conditions at an early stage. Addressing risk and protective factors in the work environment could tribute to promote occupational well-being, preventing exhaustion disorder and long-term sick leave. The risk and protective work factors could also be targets of future preventive workplace interventions among employees in the municipal sector in rural northern Sweden.

## Ethical approval

This study was approved by the Regional Ethical Review Board in Sweden, Dnr 2017/495-31.

## Informed consent

Informed consent was obtained from all participants.

## Conflict of interest

The authors report no conflict of interest.

## Funding

This study was funded by a Coordination Association of municipalities in northern Sweden.
